# Hepatitis B Virus Reactivation Increased the Risk of Developing Hepatic Failure and Mortality in Cirrhosis With Acute Exacerbation

**DOI:** 10.3389/fmicb.2022.910549

**Published:** 2022-07-07

**Authors:** Ying Zhu, Hai Li, Xianbo Wang, Xin Zheng, Yan Huang, Jinjun Chen, Zhongji Meng, Yanhang Gao, Zhiping Qian, Feng Liu, Xiaobo Lu, Yu Shi, Jia Shang, Huadong Yan, Yubao Zheng, Liang Qiao, Yan Zhang, Xiaomei Xiang, Yunjie Dan, Shuning Sun, Yixin Hou, Qun Zhang, Yan Xiong, Sumeng Li, Jun Chen, Zebing Huang, Beiling Li, Xiuhua Jiang, Sen Luo, Yuanyuan Chen, Na Gao, Chunyan Liu, Liujuan Ji, Wei Yuan, Jing Li, Tao Li, Rongjiong Zheng, Xinyi Zhou, Haotang Ren, Yi Zhou, Baoyan Xu, Rentao Yu, Wenting Tan, Guohong Deng

**Affiliations:** ^1^Department of Infectious Diseases, Southwest Hospital, Third Military Medical University (Army Medical University), Chongqing, China; ^2^Department of Gastroenterology, Ren Ji Hospital, School of Medicine, Shanghai Jiao Tong University, Shanghai, China; ^3^Center of Integrative Medicine, Beijing Ditan Hospital, Capital Medical University, Beijing, China; ^4^Department of Infectious Diseases, Institute of Infection and Immunology, Union Hospital, Tongji Medical College, Huazhong University of Science and Technology, Wuhan, China; ^5^Department of Infectious Diseases, Hunan Key Laboratory of Viral Hepatitis, Xiangya Hospital, Central South University, Changsha, China; ^6^Hepatology Unit, Department of Infectious Diseases, Nanfang Hospital, Southern Medical University, Guangzhou, China; ^7^Department of Infectious Diseases, Hubei Clinical Research Center for Precise Diagnosis and Therapy of Liver Cancer, Taihe Hospital, Hubei University of Medicine, Shiyan, China; ^8^Department of Hepatology, The First Hospital of Jilin University, Changchun, China; ^9^Department of Liver Intensive Care Unit, Shanghai Public Health Clinical Centre, Fudan University, Shanghai, China; ^10^Department of Infectious Diseases and Hepatology, The Second Hospital of Shandong University, Jinan, China; ^11^Infectious Disease Center, The First Affiliated Hospital of Xinjiang Medical University, Urumqi, China; ^12^State Key Laboratory for Diagnosis and Treatment of Infectious Diseases, Collaborative Innovation Center for Diagnosis and Treatment of Infectious Disease, The First Affiliated Hospital, Zhejiang University School of Medicine, Hangzhou, China; ^13^Department of Infectious Diseases, Henan Provincial People’s Hospital, Zhengzhou, China; ^14^Department of Hepatology, Hwamei Hospital, Ningbo No.2 Hospital, University of Chinese Academy of Sciences, Ningbo, China; ^15^Department of Infectious Diseases, The Third Affiliated Hospital of Sun Yat-Sen University, Guangzhou, China; ^16^Chongqing Key Laboratory for Research of Infectious Disease, Chongqing, China

**Keywords:** acute-on-chronic liver failure, cohort study, Hepatitis B virus reactivation, cirrhosis, mortality

## Abstract

**Background and Aims:**

Hepatitis B virus (HBV) reactivation is a serious condition and has been extensively described in chemotherapeutic immunosuppressive population. However, little is known about HBV reactivation in immunocompetent patients with chronic hepatitis B (CHB). In this study, we evaluated the prevalence and the clinical significance of HBV reactivation in CHB patients with acute exacerbations.

**Method:**

Patients were screened from two prospective multicenter observational cohorts (CATCH-LIFE cohort). A total of 1,020 CHB patients with previous antiviral treatment history were included to assess the prevalence, risk factors, clinical characteristics of HBV reactivation, and its influence on the progression of chronic liver disease.

**Results:**

The prevalence of HBV reactivation was 51.9% in CHB patients with acute exacerbations who had antiviral treatment history in our study. Among the 529 patients with HBV reactivation, 70.9% of them were triggered by discontinued antiviral treatment and 5.9% by nucleos(t)ide analogs (NUCs) resistance. The prevalence of antiviral treatment disruption and NUCs resistance in patients with HBV reactivation is much higher than that in the patients without (70.9% vs. 0.2%, and 5.9% vs. 0, respectively, both *p* < 0.001). Stratified and interaction analysis showed that HBV reactivation was correlated with high short-term mortality in cirrhosis subgroup (HR = 2.1, *p* < 0.001). Cirrhotic patients with HBV reactivation had a significantly higher proportion of developing hepatic failure (45.0% vs. 20.3%, *p* < 0.001), acute-on-chronic liver failure (ACLF; 31.4% vs. 21.8%, *p* = 0.005), and short-term death (14.0% vs. 5.9% for 28-day, and 23.3% vs. 12.4% for 90-day, both *p* < 0.001) than those without. HBV reactivation is an independent risk factor of 90-day mortality for cirrhosis patients (OR = 1.70, *p* = 0.005), as well as hepatic encephalopathy, ascites, and bacterial infection.

**Conclusion:**

This study clearly demonstrated that there was a high prevalence of HBV reactivation in CHB patients, which was mainly triggered by discontinued antiviral treatment. The HBV reactivation strongly increased the risk of developing hepatic failure, ACLF and short-term death in HBV-related cirrhotic patients, which may suggest that HBV reactivation would be a new challenge in achieving the WHO target of 65% reduction in mortality from hepatitis B by 2030.

## Introduction

Chronic hepatitis B (CHB) remains a significant global health burden, and affects an estimated 250 million people worldwide (Smith et al., 2019). CHB is a potentially life-threatening disease which is mainly caused by HBV persistence infection, and can lead to liver irreversible injury, cirrhosis. Cirrhosis typically has two phases: an asymptomatic phase (compensated cirrhosis) followed by a rapidly progressive phase (decompensated cirrhosis) signaled by the development of complications of portal hypertension and liver dysfunction, which can progress to acute decompensation (AD), or AD with multi-organ failure known as acute-on-chronic liver failure (ACLF; Moreau et al., 2013). Patients with cirrhosis are susceptible to a range of acute hepatic insults, including HBV reactivation, bacterial infection, recent alcohol intake, hepatotoxic drugs, and acute variceal bleeding. Acute hepatic insult is the main cause of hospitalization in patients with CHB in the Asian region (Sarin et al., 2019).

HBV reactivation, defined by the abrupt reappearance or rise of HBV DNA in the serum of a patient with previously inactive or resolved HBV infection, is considered as the main cause of acute hepatic insult in CHB patients in East (Sarin et al., 2019). HBV reactivation is predominantly due to the unbalanced state between host immunity and viral replication (Shi and Zheng, 2020). This event can be caused by a variety of factors including anticancer agents, immunosuppressive, biological therapies, and occur spontaneously. Previous studies suggested that the HBV reactivation rate was 25% (ranged 4%–68%) in cancer patients with previously HBV infection after chemotherapy or immunosuppressive therapy (Paul et al., 2015). Approximately 65% of these patients had disease progressed and even could reach hepatic failure, which required liver transplantation or death (Karvellas et al., 2017). A recent study from Egypt in HBsAg-positive patients who was undergoing direct-acting antivirals (DAAs) against HCV showed that 28.6% of the patients appeared HBV reactivation and only 1(10%) with liver hepatitis (El Kassas et al., 2018).

However, many previous studies were retrospective (Terrault et al., 2018) and most of them were focused on HBV reactivation in the settings of patients underwent chemotherapy or immunosuppressive therapy, therefore the data was lacking to fully describe the incidence of HBV reactivation and its related characteristics in immunocompetent patients with CHB. In the current study, we evaluated the prevalence and the clinical significance of HBV reactivation in CHB patients with acute exacerbation in a prospective multicenter cohort.

## Materials and Methods

### Study Population

**Figure 1:**
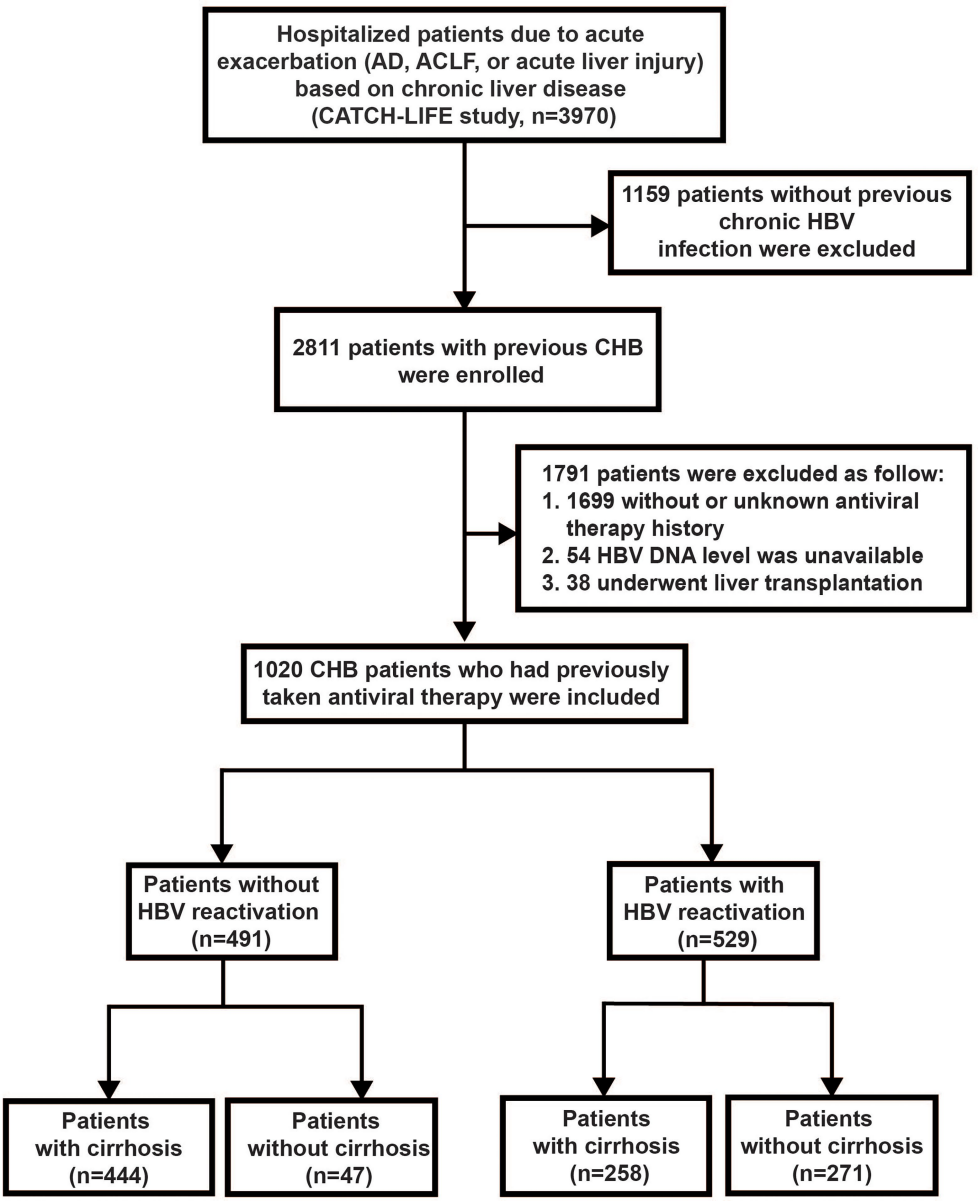
Screening, enrollment, and flow of patients. AD, acute decompensation; ACLF, acute-on-chronic liver failure; and CHB, chronic hepatitis B.

Patients were screened from two prospective multicenter observational cohorts (Investigation and Validation cohort of CATCH-LIFE study; Gu et al., 2018; Qiao et al., 2021), recruited from 15 hospitals in HBV high-endemic areas of China from January 2015 to December 2016 and July 2018 to January 2019, respectively. Briefly, there were 2600 patients with acute exacerbation of chronic liver disease in the investigation cohort and 1397 patients under the same inclusion criteria in the validation cohort. The patients with immunosuppressive therapies history were excluded at the enrollment stage of the cohort. The detailed cohort design protocol and basement characteristics of the patients were described previously (Gu et al., 2018; Qiao et al., 2021). All participants were followed up for at least 90 days by telephone call and/or clinic visit with a predesigned standard case report form (CRF) to obtained and recorded the 28-day and 90-day severe outcomes. Within these patients, we further identified the CHB patients with an antiviral treatment history as the subset to assess the influence of HBV reactivation on the progression and outcomes of disease in this study. Totally, 1,020 CHB patients with acute exacerbation who had antiviral treatment history were included in this study (Figure 1). The study was approved by the Ren Ji Hospital Ethics Committee of Shanghai Jiaotong University School of Medicine. Written informed consent were obtained from every participant or their legal surrogate.

### Definitions

Cirrhosis was diagnosed based on liver biopsy or clinical presentation with typical ultrasound or computed tomography imaging. Acute exacerbation of CHB was defined as nonmalignant CHB with AD or acute liver injury. Diagnostic criteria of AD: (1) acute development of at least one of the gastrointestinal hemorrhage, hepatic encephalopathy (HE), overt ascites or bacterial infection, or (2) jaundice [total bilirubin (TB) > 5 mg/dl] within 1 month before enrollment. Diagnostic criteria of acute liver injury: (1) serum level of alanine aminotransferase (ALT) or aspartate aminotransferase (AST) was three times higher than the upper limit of normal, or (2) serum level of TB was two times greater than the upper limit of normal within 1 week. ACLF was defined according to the EASL-CLIF criteria (Moreau et al., 2013) regardless of cirrhosis. We evaluated whether ACLF existed at admission (day 0) or ACLF newly developed at day 4, 7, 14, 21 and day 28 during hospitalization.

### HBV Reactivation Assessment and Data Collection

While the EASL-CLIF criteria did not provide recommendations for the diagnosis of HBV reactivation, the occurrence of HBV reactivation was assessed according to the recommendation (Terrault et al., 2018) of AASLD in 2018: (1) For HBsAg-positive and anti-HBc positive patients, an increase of more than 2 Log_10_ IU/ml in HBV DNA compared to baseline, or 4 Log_10_ IU/ml increase if the baseline level is not available; or (2) For HBsAg-negative and anti-HBc positive patients, HBV DNA is detectable or reappearance of HBsAg (Terrault et al., 2018); and further limited as (3) presenting with a definite antiviral treatment history more than 6 months, to decrease the heterogeneity of HBV reactivation population in this study. The data of clinical characteristics, laboratory tests and complications were obtained with standardized case report forms (CRF) from the medical records of each patient. All data were checked by a physician and two clinical assistants.

### Statistical Analysis

The data were analyzed with SPSS software (version 25.0, SPSS Inc., Chicago, United States). Continuous variables are presented as mean ± standard deviation (SD) or median with interquartile range (IQR) while comparisons were performed between groups by Student’s *t*-test or Mann–Whitney *U* test, respectively. Categorical variables are presented as proportions while comparisons were performed between groups using the *χ*^2^ test or Fisher’s exact test where appropriate. Univariate comparisons were performed using the Kruskal–Wallis test. Multivariable COX hazard analysis was applied to determine the independent factors for 90-day mortality. Survival rates were estimated by Kaplan–Meier analysis and Log-rank test. Statistical significance was set at *p* < 0.05 of two-tailed.

## Results

### Prevalence and Risk Factors of HBV Reactivation in CHB Patients With Acute Exacerbation

Out of the total 2811 CHB patients with acute exacerbation from the CATCH-LIFE cohort, we identified 1,020 subjects with antiviral therapy history and available HBV DNA level to set as sub-population in this study (Figure 1). Among these 1,020 patients, 529 of them experienced HBV reactivation and 491 of them did not, the prevalence of HBV reactivation in CHB patients with previous antiviral therapy history was 51.9%, while that was 18.8% (529/2,811) in the total 2,811 CHB patients with acute exacerbation regardless of antiviral history. The baseline of 1,020 patients are shown in Supplementary Table 1.

**Figure 2:**
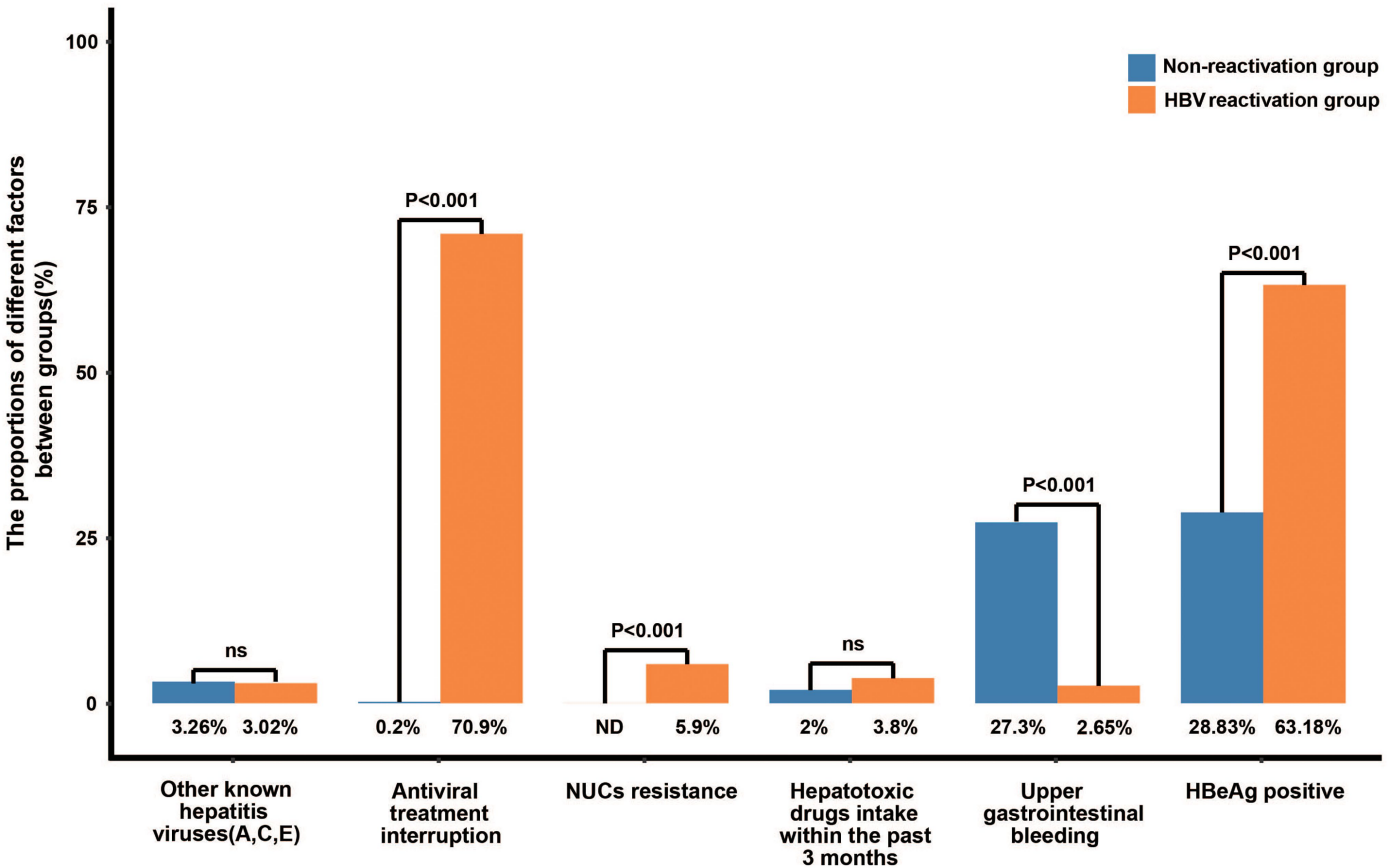
Risk factors of HBV reactivation in CHB patients.

Among the 529 patients with HBV reactivation, 375 patients (70.9%) were triggered by discontinued antiviral treatment and 31 patients (5.9%) by nucleos(t)ide analogs (NUCs) resistance (Figure 2). The prevalence of antiviral treatment disruption and NUCs resistance in patient with HBV reactivation is much higher than that in the patients without HBV reactivation (70.9% vs. 0.2%, and 5.9% vs. 0, respectively, both *p* < 0.001, Figure 2). The ratio of the other hepatitis virus (HAV, HCV, and/or HEV) co-infection and the hepatotoxic drugs taken within the past 3 months were similar between the two groups (3.02% vs. 3.26%, and 3.8% vs. 2.0%, respectively, both *p* > 0.05). The events of bacterial infection (15.3% vs. 21.4%, *p* = 0.012), ascites (35.4% vs. 55%, *p* < 0.001), and gastrointestinal bleeding (2.7% vs. 27.3%, *p* < 0.001) were less frequently happened in patients with HBV reactivation than those without, which may due to the higher proportion of cirrhosis in non-HBV reactivation group (90.4% vs. 48.8%, *p* < 0.001; Figure 2; Supplementary Table 1). Notably, the rate of serum HBeAg-positive was significantly higher in patients with HBV reactivation than that without (63.2% vs. 28.8%, *p* < 0.001, Figure 2; Supplementary Table 1).

### Stratified and Interaction Analysis Showed That HBV Reactivation Was Correlate With Critical Outcome in Cirrhosis Subgroup

**Figure 3:**
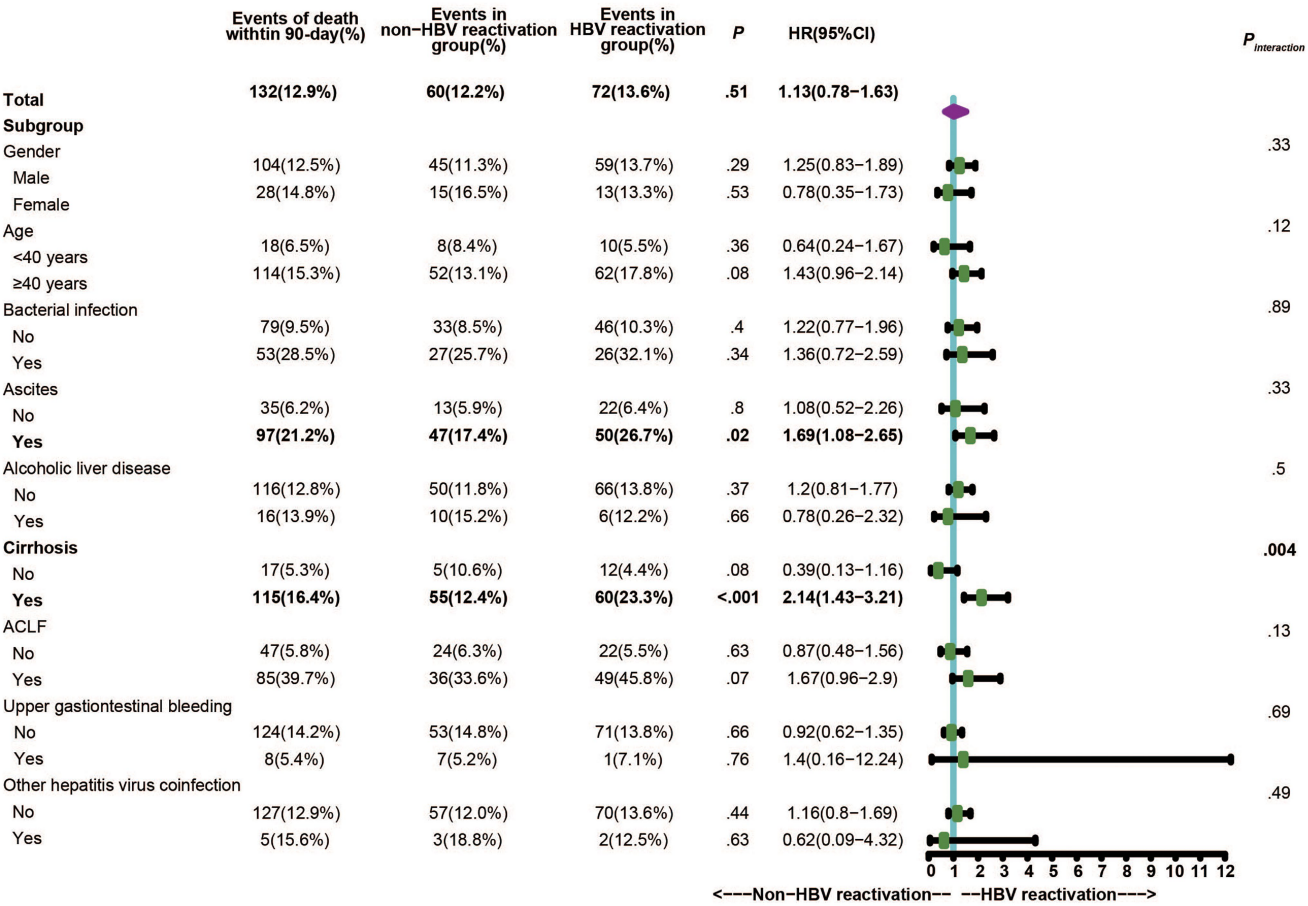
Stratified and interaction analysis to identify the subgroup that interacted with HBV reactivation on the critical outcome of CHB.

Within the 1,020 patients, no significant difference was observed for the 28-day and 90-day mortality between CHB patients with HBV reactivation and that without (*p* > 0.05). Considering the proportion of underlying cirrhosis was unbalance between two groups (Supplementary Table 1) which may resulted in bias and confusion, we further performed stratified analysis by dividing individuals into subgroups to evaluate and control confounding factors. We applied 90-day mortality as the event to compare the difference between CHB patients with or without HBV reactivation within these subgroups. As shown in Figure 3, there were 133 events of death within 90-days totally, which was 72 in HBV reactivation group and 61 in non-HBV reactivation group [13.6% vs. 12.4%, *p* = 0.57, hazard ratio (HR) = 1.11]. Remarkably, in cirrhosis subgroup the events of death within 90-days in CHB patients with HBV reactivation were significantly higher than that in CHB patients without HBV reactivation (23.3% vs. 12.4%, *p* < 0.001, HR = 2.10, 95% CI = 1.40–3.14, Figure 3). What is more, a significant interaction between HBV reactivation and cirrhosis (P_interaction_ = 0.004) was observed by the test for interaction under the Cox proportional hazards model. It suggested that the combination of HBV reactivation and cirrhosis strongly increased the risk of short-term death. No similar significance or interaction was observed in non-cirrhotic subgroup or any other subgroups (*p* > 0.05).

### Characteristics and Influence of HBV Reactivation in Cirrhotic CHB Patients With Acute Exacerbation

In order to explore the influence of HBV reactivation on the disease progression of cirrhosis, we further compared the characters and the short-term mortality between cirrhotic patients with and without HBV reactivation in the cirrhosis subgroup.

All 702 cirrhotic patients with acute exacerbation were included in this analysis and the baseline characteristics are shown in Table 1 (while the baseline of 318 non-cirrhotic patients is shown in Supplementary Table 3). Compared to the patients without HBV reactivation, HBV reactivation patients were a little bit younger (49.0 ± 10.0 vs. 50.6 ± 10.5 years, *p* = 0.04) and had a significantly lower incidence of gastrointestinal bleeding (5.4% vs. 30.2%, *p* < 0.001), while there was no significant difference in the incidence of bacterial infection (*p* = 0.92), ascites (*p* = 0.31), and others hepatitis virus co-infection (*p* = 0.84) between the two groups. However, cirrhotic patients with HBV reactivation had a significantly higher ALT, AST, alkaline phosphatase (ALP) and γ-glutamyltransferase (γ-GT) level (both *p* < 0.001), as well as bilirubin level and international normalized ratio (INR; both *p* < 0.001), than the non-HBV reactivation group, which suggested much more heavy liver injury when cirrhosis patient suffered from HBV reactivation. Overall, 45.0% of HBV reactivation patients developed liver failure, which was significantly higher than that of 20.3% in the non-HBV reactivation cirrhotic patients (*p* < 0.001, Table 1).

**Table 1:** Characteristics of patients with cirrhosis between HBV reactivation and non-reactivation.

	Total (*n* = 702)	Non-HBV reactivation (*n* = 444)	HBV reactivation (*n* = 258)	*p*
**Demographic, ***n*** (%)**				
Male sex	565 (80.5)	360 (81.1)	205 (79.5)	0.60
Age, *y*, mean ± SD	50.0 ± 10.4	50.6 ± 10.5	49.0 ± 10.0	0.04
Alcohol consumption	93 (13.3)	61 (13.7)	32 (12.4)	0.62
Other hepatitis virus	23 (3.3)	15 (3.4)	8 (3.1)	0.84
**Complication, ***n*** (%)**				
Bacterial infection	166 (23.7)	98 (22.1)	68 (26.4)	0.92
Ascites	429 (61.1)	265 (59.7)	164 (63.6)	0.31
Gastrointestinal bleeding	148 (21.1)	134 (30.2)	14 (5.4)	<0.001
Hepatic encephalopathy				0.72
Grade I	27 (3.9)	19 (4.3)	8 (3.1)	
Grade II	28 (4.0)	19 (4.3)	9 (3.5)	
Grade III	6 (0.9)	4 (0.9)	2 (0.8)	
Grade IV	5 (0.7)	2 (0.5)	3 (1.2)	
**HBV markers**				
HBeAg positive, n/N (%)	226/624 (36.2)	102/394 (25.9)	124/230 (53.9)	<0.001
HBeAb positive, n/N (%)	180/625 (28.8)	135/393 (34.4)	45/232 (19.4)	<0.001
HBV DNA, log_10_ IU/ml, median (IQR)	2.7 (1.7–5.0)	2.0 (1.2–2.7)	5.7 (4.6–6.8)	<0.001
**Laboratory test, median (IQR)**				
Alanine transaminase, U/L	49.1 (25.2–158.0)	31.1 (20.0–55.4)	172.0 (72.6–456.0)	<0.001
Aspartate transaminase, U/L	67.0 (35.0–169.0)	43.0 (28.0–81.0)	182.0 (87.1–386.0)	<0.001
Alkaline phosphatase, U/L	114.0 (80.0–161.0)	98.2 (66.8–145.0)	136.0 (108–171.0)	<0.001
γ-glutamyltransferase, U/L	48.8 (24.0–96.2)	32.0 (18.5–66.0)	84.0 (49.0–136.0)	<0.001
Albumin, g/L	31.0 (27.1–34.7)	31.1 (27.1–35.0)	30.8 (27.3–34.4)	0.43
Total bilirubin, mg/dL	3.9 (1.5–14.1)	2.6 (1.1–9.6)	10.0 (3.3–21.0)	<0.001
White blood cell, 10^9^/L	4.6 (3.2–6.6)	4.4 (2.9–6.5)	5.0 (3.7–6.9)	0.016
Platelet, 10^9^/L	66.0 (44.0–101.0)	63.0 (42.0–99.0)	73.0 (51.0–106.0)	0.71
International normalized ratio	1.5 (1.3–1.9)	1.5 (1.3–1.8)	1.6 (1.4–2.1)	<0.001
Creatinine, mg/dl	0.8 (0.7–1.0)	0.8 (0.7–1.0)	0.8 (0.6–1.0)	0.35
Sodium, mmol/L	138 (134–140)	138 (134–140)	138 (135–140)	0.52
**Organ failures, ***n*** (%)**				
Liver failure	206 (29.3)	90 (20.3)	116 (45.0)	<0.001
Coagulation failure	88 (11.4)	46 (10.4)	34 (13.2)	0.26
Kidney failure	22 (3.1)	15 (3.4)	7 (2.7)	0.63
Cerebral failure	11 (1.6)	6 (1.4)	5 (1.9)	0.55
**Adverse outcome, ***n*** (%)**				
28-day death	62 (8.8)	26 (5.9)	36 (14.0)	<0.001
90-day death	115 (16.4)	55 (12.4)	60 (23.3)	<0.001
Diagnosed as ACLF within 28 days^*^	178 (25.4)	97 (21.8)	81 (31.4)	0.005

* ACLF was evaluated at admission (day 0) and day 4, 7, 14, 21, and 28 during hospitalization.

SD, standard deviation and IQR, interquartile range. The identification of organ failure was based on CLIF-OF (Moreau et al., 2013).

**Figure 4:**
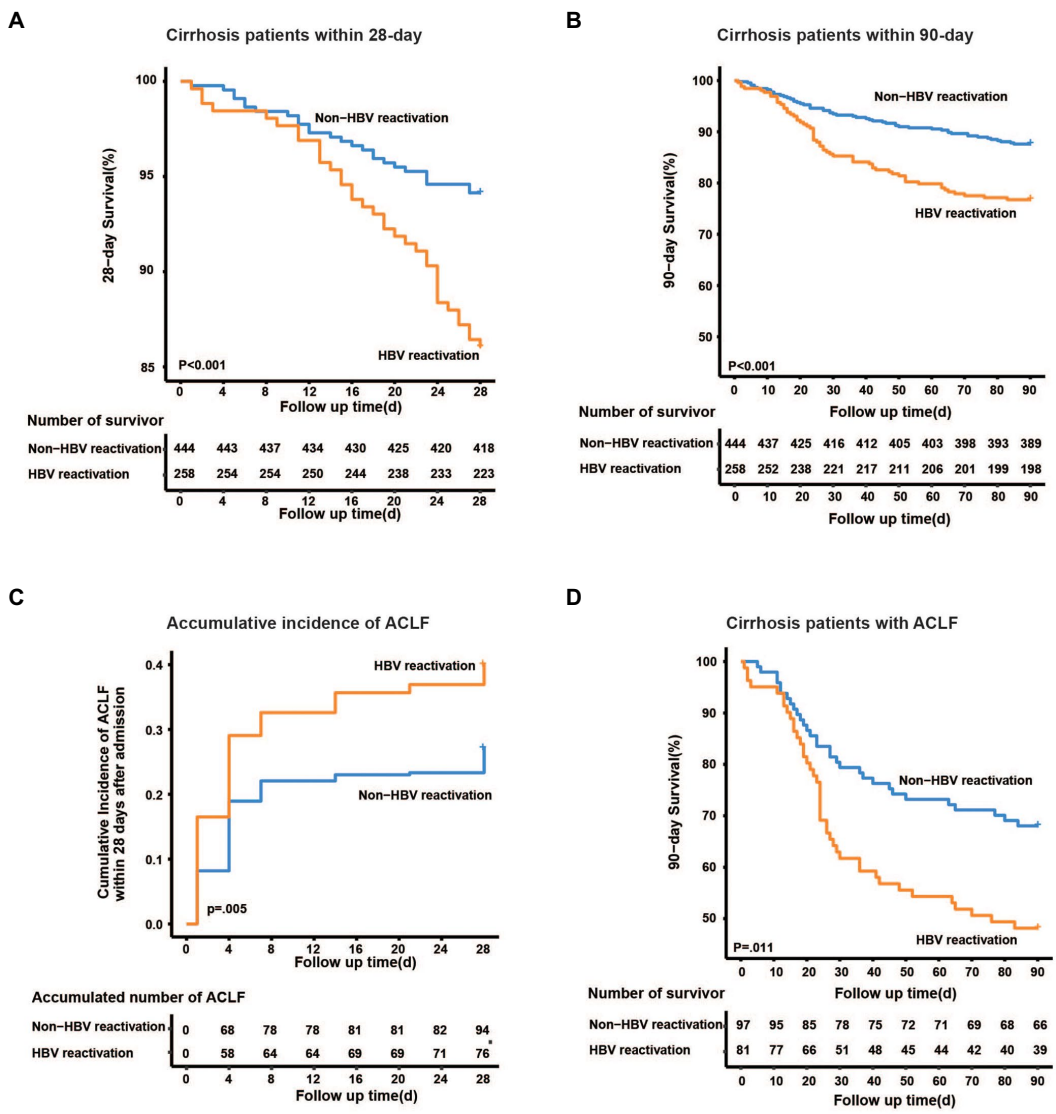
Kaplan–Meier analysis of survival rate and accumulative developing of ACLF. Kaplan–Meier curves are shown for the cumulative incidence of 28-day death **(A)**, 90-day death **(B)** and developing of ACLF **(C)** in cirrhotic patients with and without HBV reactivation. **(D)** Cumulative 90-day mortality in ACLF patients between groups.

Furthermore, the 28-day mortality was significantly higher in HBV reactivation patients than that in the non-HBV reactivation patients (14.0% vs. 5.9%, *p* < 0.001, Figure 4A), as well as 90-day mortality (23.3% vs. 12.4%, *p* < 0.001, Figure 4B), which suggested a worse outcome when cirrhosis patient suffered from HBV reactivation.

### HBV Reactivation Increased the Risk of Developing ACLF in Cirrhosis Patients and the Mortality of ACLF

To discover whether HBV reactivation plays a role in the development of liver failure in cirrhotic patients with acute exacerbation, we further analyzed the accumulative incidence and characteristics of ACLF. Among the 702 cirrhotic patients, 178 of them were diagnosed as ACLF within 28 days after admission. The cumulative incidence of ACLF within 28 days was significantly higher in cirrhotic patients with HBV reactivation than that without HBV reactivation (31.4% vs. 21.8%, *p* = 0.005, Figure 4C), which may suggest that HBV reactivation increased the risk of developing ACLF in cirrhosis patient with acute exacerbation.

Notably, among the 178 patients who developed ACLF, the incidence of hepatic failure was significantly higher when patients underwent HBV reactivation (87.7% vs. 53.6%, *p* < 0.001; Supplementary Table 2), while that of coagulation failure and extrahepatic organ failure (kidney, cerebral or lung) seemed to be similar between HBV reactivation ACLF and non-HBV reactivation ACLF (*p* > 0.05). For those cirrhotic ACLF patients without HBV reactivation, more precipitant events of alcohol consumption and gastrointestinal bleeding were observed (*p* = 0.039 and *p* = 0.002, respectively; Supplementary Table 2).

Furthermore, for those 178 ACLF patients, the 28-day and 90-day mortality was much higher in those with HBV reactivation than those without HBV reactivation (35.8% vs. 18.6%, *p* = 0.009; 51.9% vs. 33.0%, *p* = 0.011, respectively, Figure 4D; Supplementary Table 2), however similar difference was not observed in non-ACLF patients (10.2% vs. 6.9%, *p* = 0.19).

### HBV Reactivation Was an Independent Risk Factor of 90-Day Mortality for Cirrhosis Patients

To discover the risk factors of mortality for cirrhosis patients with acute exacerbation and whether HBV reactivation have an independent influence on the short-term death, we performed the univariate and multivariate analysis among the 702 cirrhotic patients. The results of the univariate analysis are showed in Table 2. HBV reactivation [odds ratio (OR) = 2.01, 95% CI = 1.40–2.90, *p* < 0.001] played a role in the 90-day mortality, as well as bacterial infection (OR = 2.51, 95% CI = 1.73–3.63, *p* < 0.001), ascites (OR = 2.75, 95% CI = 1.74–4.34, *p* < 0.001) and HE grades III-IV (OR = 5.22, 95% CI = 2.29–11.89, *p* < 0.001).

**Table 2:** Univariate and multivariate analysis of the risk factors of the 90-day death in cirrhosis patients.

Variable	Univariate analysis			Multivariate analysis		
	*p*	OR	95% CI	*p*	AOR	95% CI
**Risk factors**						
Male sex	0.58	0.88	0.57–1.37			
Age	0.28	1.01	0.99–1.03			
Bacterial infection	<0.001	2.51	1.73–3.63	0.001	1.98	1.34–2.92
Ascites	<0.001	2.75	1.74–4.34	0.002	2.09	1.30–3.33
HE grades III-IV	<0.001	5.22	2.29–11.89	<0.001	4.71	2.01–11.05
Alcoholic liver disease	0.96	0.99	0.57–1.7			
Gastrointestinal bleeding	<0.001	0.26	0.13–0.53	0.041	0.48	0.24–0.97
HBV reactivation	<0.001	2.01	1.40–2.9	0.005	1.70	1.17–2.49
Other viral hepatitis	0.67	0.78	0.25–2.45			
Sepsis	<0.001	4.72	2.30–9.70	0.006	2.84	1.35–5.97
**Predict markers**						
Alanine transaminase	0.011	1.00	1.00–1.00			
Aspartate transaminase	0.003	1.00	1.00–1.00			
Alkaline phosphatase	<0.001	1.01	1.00–1.01			
γ-Glutamyltransferase	0.72	1.00	1.00–1.002			
Albumin	0.001	0.95	0.92–0.98	<0.001	0.93	0.89–0.97
Total bilirubin	<0.001	1.08	1.07–1.09	<0.001	1.07	1.05–1.09
International normalized ratio	<0.001	2.92	2.41–3.55	<0.001	1.68	1.29–2.19
Creatinine	0.017	1.19	1.03–1.38			
White blood cell	<0.001	1.06	1.04–1.09			
Platelet	0.94	1.00	1.00–1.00			
Log_10_ HBV DNA	<0.001	1.17	1.08–1.26			

OR, odds ratio; AOR, adjusted odds ratio; CI, confidence interval; and HE, hepatic encephalopathy.

Furthermore, in the multivariate analysis we included the variables with values of *p* less than 0.05 in the univariate analysis to identify whether HBV reactivation perform an independent influence on the severe outcome of cirrhosis. The results showed that HBV reactivation (OR = 1.70, 95% CI = 1.17–2.49, *p* = 0.005) was really an independent risk factor of 90-day mortality for cirrhosis patients besides the risk factors bacterial infection (OR = 1.98, *p* = 0.001), ascites (OR = 2.09, *p* = 0.002), HE grades III-IV (OR = 4.71, *p* < 0.001), and sepsis (OR = 2.84, *p* = 0.006), all details are listed in Table 2.

However, serum level of HBV DNA was not a predictive factor for the 90-martality (OR = 1.08, 95% CI = 1.00–1.18, *p* = 0.13). Among the laboratory variables listed in Table 2, only baseline level of INR was a significant strong predictor for the mortality of cirrhosis patient with acute exacerbation (OR = 1.68, 95% CI = 1.29–2.19, *p* < 0.001) rather than liver enzymes (ALT, AST, ALP, Ƴ-GT), bilirubin, albumin, creatinine, white blood cells count or platelet.

## Discussion

It was reported that HBV reactivation could occur spontaneously or conditionally (Shi and Zheng, 2020), especially it was likely to occur when chemotherapeutic immunosuppressive agents were given for patients with cancer, immunity disease and organ transplantation (Paul et al., 2015; Karvellas et al., 2017), and when DAAs treatment was given for patients with HBV/HCV coinfection (El Kassas et al., 2018). HBV reactivation was a serious condition which could cause significantly higher mortality in these patients. However, little is known about the reactivation of HBV in widely immunocompetent patient with CHB. Here we provide new sight on this issue.

In our present study, the prevalence of HBV reactivation was 51.9% in CHB patients with acute exacerbations who had an antiviral treatment history. It seems that the HBV reactivation becomes a new remarkable burden after two decades of effort on widespread antiviral treatment of hepatitis B in China. Although the World Health Organization (WHO) has set the global targets to eliminate hepatitis B as a public health threat by 2030 through vaccination, screening and treatment (WHO, 2016), researchers still worry about that the elimination goal is highly unlikely to be reached (Cox et al., 2020) given the low average diagnosis rate of hepatitis B, which is only about 8% globally (Thomas, 2019). From the result of this study, HBV reactivation in CHB patients may be another factor would prevent the reach of the global goal and need to be concerned more.

Antiviral drug withdrawal or interruption was the predominant reason of the HBV reactivation in our present study. Among the 529 patients with HBV reactivation, 70.9% of them were triggered by cessation of antiviral treatment. Although both guidelines of APASL, EASL, and AASLD have put forward elaborate recommendations on NUCs treatment and withdrawal, it is still common in clinical practice that patients have poor adherence and stop NUCs treatment under various reasons, with a reported treatment adherence about 46.4%–74.6% (Ford et al., 2018; Abu-Freha et al., 2020), which may results in virological breakthrough and increasement of mortality (Abu-Freha et al., 2020). Taken together, it suggests that more attention should be paid on the adherence of antiviral treatment in CHB patients to avoid HBV reactivation and its related critical outcomes.

In our study, stratified and interaction analysis showed that HBV reactivation was strongly increased the risk of short-term death in cirrhosis subgroup. The mortality was 14.0% for 28-day and 23.3% for 90-day in cirrhotic patients with HBV reactivation, which was significantly higher than that of 5.9% and 12.4%, respectively, in cirrhotic patients without HBV reactivation. There is limited research or cohort data about the mortality in immunosuppressive-therapy-induced HBV reactivation, it was reported to be 3.7% in HBsAg-positive patients and 0% in HBsAg-negative patients who received cytotoxic therapy for malignant lymphoma in an earlier article (Lok et al., 1991), the mortality is less than that of cirrhotic patients from our study which may due to the underlying conditions. Previous case reports described the HBV reactivation death due to liver failure despite the introduction of lamivudine at the onset of HBV reactivation (Lok et al., 1991; Gwak et al., 2007). To discover the possible reason of high mortality in cirrhosis with HBV reactivation, we further found that HBV reactivation strongly increased the risk of developing liver injury, liver failure and ACLF in cirrhotic CHB patients. ACLF is a critical syndrome distinguished from AD, characterized by high-grade inflammation and high 28-day mortality (Moreau et al., 2013). The cumulative incidence of ACLF within 28 days in our study was significantly higher in cirrhotic patients with HBV reactivation and consequently a higher 28-day and 90-day mortality than that without HBV reactivation, which suggested that HBV reactivation could increase the risk of developing ACLF and related death in cirrhosis patient with acute exacerbation. To discover whether the selection of different ACLF criteria could make a difference to the conclusion, we further analyzed the accumulative incidence of ACLF in our cohort under APASL (Sarin et al., 2019) and COSSH criteria (Wu et al., 2018), the results (data were not shown) was consistent with this study. As we and other studies have reported, liver and coagulation failures were the two most common types of organ failure in patients with HBV-related ACLF (Tan et al., 2018). Among the 178 patients who developed ACLF in this study, the incidence of hepatic failure was up to 87.7% in ACLF patients with HBV reactivation, which was significantly higher than that without, while that of extrahepatic organ failure (kidney, cerebral or lung) was similar between two groups.

In addition, we found that HBV reactivation was an independent risk factor for the 90-days mortality of CHB patients with cirrhosis, remained a high risk (1.7-fold) after adjusting by other factors as age, sex, bacterial infection, ascites, hepatic encephalopathic, alcoholic assumption, gastrointestinal bleeding, sepsis and other viral hepatitis. Previous study reported that half of HBeAg-positive patients had the chance of HBeAg seroconversion within 3 months after hepatitis flare (Yuen et al., 2003). In a proof-of-concept study, stopping antiviral therapy in non-cirrhotic patients with HBeAg-negative CHB resulted in HBV rebound and subsequent significantly HBsAg decline, with 20% HBsAg loss in 48-week follow-up (Höner Zu Siederdissen et al., 2016), suggesting the immune activation in those patients and a potential therapeutic strategy to accelerate HBsAg loss and functional cure (Höner Zu Siederdissen et al., 2016; van Bommel and Berg, 2021). However, this antiviral-stopping therapeutic strategy seems to be not feasible in cirrhosis patients considering the high incidence of HBV reactivation and its related high risk of liver failure and death from the data of our present study.

Similar correlation between short-term death and HBV reactivation was not observed in non-cirrhotic subgroup. Adversely, it showed a lower (4.4%) but not significant mortality in non-cirrhotic patients with HBV reactivation than that without (10.6%). One of the possible explanations might be that the non-cirrhosis patient in our population was younger (median age 39.8 years, data were shown in Supplementary Table 3). Moreover, there were only 47 non-cirrhotic patients without HBV reactivation, the volume was too small which might lead to bias when calculated the mortality. Thus, more evidence is need to be carried out to clarify whether HBV reactivation plays a role or not in non-cirrhotic patients.

Our study has several strengths. Firstly, in this prospective multicenter observational cohort, the HBV reactivation were observed in common CHB population who were not under an immunosuppressive condition, which provides a better understanding of the association between HBV reactivation and the progression of disease in HBV-related cirrhosis patients. Secondly, participants in our study belong to 15 hepatology or infectious disease centers in a high-endemic area of HBV, and the prospective multicenter design of the cohort with low rate of loss in follow-up, which ensured that the results were representative and reliable. Thirdly, it is an evidence-based data showed a high prevalence rate of HBV reactivation which major duo to antiviral treatment withdrawal in general CHB patients. Finally, it is a unique and evidence-based study in demonstrating the independent correlation between HBV reactivation and critical outcomes in cirrhotic patients with acute exacerbation.

This study has the following limitations. Untreated patients were excluded in this study due to lacked data of medical history or unclear antiviral treatment history. HBV flares in these patients will deserve attention in the future studies. Furthermore, despite we showed that antiviral treatment withdrawal accounted for the most of the HBV reactivation in CHB, however the details of antiviral drugs (NUCs or IFNs) and the discontinuation reason (stopped under the guidance of doctor when met the endpoint or stopped themselves due to personal reason) were not distinguished in this study. Additionally, it is hard to obtained the baseline level of HBV DNA before reactivation since most of them would not visit clinic or hospital before hepatitis flare. However, we had applied the widely-accepted AASLD definition (Terrault et al., 2018) which had a definite criterion when baseline HBV DNA level is not available, to maximum precision of the study as possible. Finally, we were not able to correlate the HBV reactivation with the clinical outcome in non-cirrhotic patients due to our limited number of patients.

In conclusion, the HBV reactivation in CHB was highly prevalent and mainly triggered by antiviral treatment withdrawal. The HBV reactivation could strongly increase the risk of developing liver injury, hepatic failure, ACLF and short-term death in cirrhosis patients, which may suggest that HBV reactivation would be a new challenge in achieving the WHO target (WHO, 2016) of 65% reduction in mortality from hepatitis B by 2030. Therefore, the antiviral-stopping therapeutic strategy, which aim to activate immune response and accelerate HBsAg loss, was not feasible in cirrhosis patients considering the high incidence of HBV reactivation and its related high risk of liver failure and death from the data of our present study.

## Transparency Statement

The lead authors affirm that the manuscript is an honest, accurate, and transparent account of the study being reported; that no important aspects of the study have been omitted; and that any discrepancies from the study as planned have been explained.

## Data Availability Statement

The original contributions presented in the study are included in the article/Supplementary Material, and further inquiries can be directed to the corresponding authors.

## Ethics Statement

The studies involving human participants were reviewed and approved by the Ren Ji Hospital Ethics Committee of Shanghai Jiaotong University School of Medicine. The patients/participants provided their written informed consent to participate in this study.

## Author Contributions

HL, JiC, WT, and GD obtained the funding. HL, WT, and GD designed the study. YZu, HL, XW, XZe, YHu, JiC, ZM, YG, ZQ, FL, XL, YS, JS, HY, YZe, LQ, YZa, XX, YD, SS, YHo, QZ, YX, SLi, JuC, ZH, BL, XJ, SLu, YC, NG, CL, LJ, WY, JL, TL, RZ, XZo, HR, YZo, BX, WT, and GD screened, enrolled, and clinically managed all the participated patients and collected the data. YZu, RY, and WT performed the statistical analysis. YZu, WT, and GD drafted the manuscript. GD and WT contributed to the critical revision of the manuscript for important intellectual content. All authors contributed to the article and approved the submitted version.

## Funding

This work was partly supported by the National Natural Science Foundation of China (81900579 and 81930061); the National Science and Technology Major Project (2018ZX10723203 and 2018ZX10732202); and the Chongqing Natural Science Foundation (CSTC2019jcyj-zdxmX0004). The funders of the study had no role in study design, data collection, analysis, data interpretation, or writing of the paper. The corresponding author had full access to all the data in the study and had final responsibility for the decision to submit for publication.

## Conflict of Interest

The authors declare that the research was conducted in the absence of any commercial or financial relationships that could be construed as a potential conflict of interest.

## Publisher’s Note

All claims expressed in this article are solely those of the authors and do not necessarily represent those of their affiliated organizations, or those of the publisher, the editors and the reviewers. Any product that may be evaluated in this article, or claim that may be made by its manufacturer, is not guaranteed or endorsed by the publisher.

## Abbreviations

CHB: Chronic hepatitis B
HBV: Hepatitis B virus
AD: Acute decompensation
UGIB: Upper gastrointestinal bleeding
ALT: Alanine aminotransferase
AST: Aspartate aminotransferase
ALP: Alkaline phosphatase
γ-GT: Gamma-glutamyltransferase
TBIL: Total bilirubin
ACLF: Acute-on-chronic liver failure
NUCs: Nucleos(t)ide analogs
Cr: Creatinine
WBC: White blood cell
INR: International normalized ratio
