# Supplementary material for: Hepatitis B Virus Reactivation Increased the Risk of Developing Hepatic Failure and Mortality in Cirrhosis With Acute Exacerbation
Source: Front Microbiol. 2022 Jul 7;13:910549. doi: 10.3389/fmicb.2022.910549 (PMC9300993; doi:10.3389/fmicb.2022.910549)
Supplement: Supplementary file 1 [file Data_Sheet_1.docx]

**Supplementary Table 1**. Baseline characteristics of 1020 CHB patients with acute exacerbation between HBV reactivation and non-reactivation.

|  | Total  n=1020 | Non-reactivation  n=491 | HBV reactivation  n=529 | P value |
| --- | --- | --- | --- | --- |
| **Demographic, n (%)** |  |  |  |  |
| Male sex | 831 (81.5) | 400 (81.5) | 431 (81.5) | >0.99 |
| Age, *y*, mean ± SD | 47.0 ± 11.5 | 49.7 ± 10.9 | 44.4 ± 11.4 | <0.001 |
| Alcohol consumption | 115 (11.3) | 66 (13.4) | 49 (9.3) | 0.04 |
| Other hepatitis virus | 32 (3.1) | 16 (3.3) | 16 (3.0) | 0.83 |
| Cirrhosis | 702 (68.8) | 444 (90.4) | 258 (48.8) | <0.001 |
| **Complication, n (%)** |  |  |  |  |
| Bacterial infection | 186 (18.2) | 105 (21.4) | 81 (15.3) | 0.012 |
| Ascites | 457 (44.8) | 270 (55.0) | 187 (35.4) | <0.001 |
| Gastrointestinal bleeding | 148 (14.5) | 134 (27.3) | 14 (2.7) | <0.001 |
| Hepatic encephalopathy |  |  |  | 0.069 |
| Grade I | 32 (3.1) | 21 (4.3) | 11 (2.1) |  |
| Grade II | 31 (3.0) | 20 (4.1) | 11 (2.1) |  |
| Grade III | 6 (0.6) | 4 (0.8) | 2 (0.4) |  |
| Grade IV | 6 (0.6) | 3 (0.6) | 3 (0.6) |  |
| **HBV markers** |  |  |  |  |
| HBeAb positive, n/N (%) | 245/915 (26.8) | 143/436 (32.8) | 102/479 (21.3) | <0.001 |
| HBeAg positive, n/N (%) | 428/915 (46.8) | 126/437 (28.8) | 302/478 (63.2) | <0.001 |
| HBV DNA, *log_10_ IU/ml*, median (IQRs) | 3.93 (2.0-6.2) | 2.00 (1.3-2.7) | 6.0 (5.0-7.2) | <0.001 |
| **Lab test, median (IQR)** |  |  |  |  |
| Alanine transaminase, *U/L* | 105 (34.0-448) | 34.9 (21.0-71.5) | 352 (132-806) | <0.001 |
| Aspartate transaminase, *U/L* | 112 (44.9-297) | 47.0 (29.0-95.2) | 251 (120-525) | <0.001 |
| Alkaline phosphatase, *U/L* | 119 (85-162) | 102 (69-151) | 132 (101-167) | <0.001 |
| γ-glutamyltransferase, *U/L* | 72.0 (32.0-137) | 37.0 (20.0-77.1) | 106 (62.2-169) | <0.001 |
| Albumin, *g/L* | 33.0 (28.8-38.1) | 31.6 (27.3-35.5) | 34.6 (30.2-39.3) | <0.001 |
| Total bilirubin, *mg/dL* | 3.8 (1.6-13.1) | 2.7 (1.2-10.0) | 5.7 (2.1-15.7) | <0.001 |
| White blood cell,*10^9^/L* | 4.8 (3.6-6.6) | 4.5 (3.0-6.6) | 5.1 (4.0-6.5) | 0.049 |
| Platelet, *10^9^/L* | 86.0 (54.0-134) | 68.0 (44.0-106) | 106.0 (68.0-153) | <0.001 |
| International normalized ratio | 1.4 (1.2-1.8) | 1.5 (1.3-1.8) | 1.38 (1.15-1.77) | 0.22 |
| Creatinine, *mg/dl* | 0.8 (0.7-1.0) | 0.9 (0.7-1.0) | 0.8 (0.7-0.9) | 0.09 |
| Sodium, *mmol/L* | 138 (135-141) | 138 (134-140) | 139 (136-141) | 0.009 |
| **Organ failures, n (%)** |  |  |  |  |
| Liver failure | 291 (28.5) | 107 (21.8) | 184 (34.8) | <0.001 |
| Coagulation failure | 88 (8.6) | 49 (10.0) | 39 (7.4) | 0.14 |
| Kidney failure | 23 (2.3) | 15 (3.1) | 1.5 (1.5) | 0.10 |
| Cerebral failure | 12 (1.2) | 7 (1.4) | 5 (0.9) | 0.48 |
| Lung failure | 2 (0.2) | 0 (0) | 2 (0.4) | 0.17 |
| **Adverse Outcome, n (%）** |  |  |  |  |
| 28-day death | 70 (6.9) | 29 (5.9) | 41 (7.8) | 0.24 |
| 90-day death | 132 (12.9) | 60 (12.2) | 72 (13.6) | 0.57 |
| Diagnosed as ACLF within 28d | 214 (21.0) | 107 (21.8) | 107 (20.2) | 0.54 |

SD, standard deviation; IQR, interquartile range; Other hepatitis virus means co-infection with hepatitis A virus, hepatitis C virus or hepatitis E virus.

**Supplementary Table 2.** Precipitants, organ failure and outcomes of ACLF patients with HBV reactivation

|  | **Total n=178** | **Non-HBV reactivation n=97** | **HBV reactivation n=81** | **P value** |
| --- | --- | --- | --- | --- |
| **Demographic** |  |  |  |  |
| Male sex, n (%) | 149 (83.7) | 80 (82.5) | 69 (85.2) | 0.63 |
| Age, *y*, median (IQR) | 49.0 (42.5-56.0) | 51.5 (45.0-58.0) | 47.0 (42.0-52.9) | 0.028 |
| **Precipitation and complication, n (%)** | |  |  |  |
| Jaundice | 140 (78.7) | 64 (66) | 76 (93.8) | <0.001 |
| Alcohol consumption | 26 (14.6) | 19 (19.6) | 7 (8.6) | 0.039 |
| Other hepatitis virus | 4 (2.2) | 3 (3.1) | 1 (1.2) | 0.41 |
| Bacterial infection | 59 (33.1) | 31 (32) | 28 (34.6) | 0.71 |
| Ascites | 123 (69.1) | 69 (71.1) | 54 (66.7) | 0.52 |
| Gastrointestinal bleeding | 21 (11.8) | 18 (18.6) | 3 (3.7) | 0.002 |
| Hepatic encephalopathy | 36 (20.2) | 21 (21.6) | 15 (18.5) | 0.61 |
| **Organ failure, n (%)** |  |  |  |  |
| Liver failure | 123 (69.1) | 52 (53.6) | 71 (87.7) | <0.001 |
| Coagulation failure | 55 (30.9) | 29 (29.9) | 26 (32.1) | 0.75 |
| Kidney failure | 21 (11.8) | 14 (14.4) | 7 (8.6) | 0.23 |
| Cerebral failure | 5 (2.8) | 2 (2.1) | 3 (3.7) | 0.51 |
| Lung failure | 0 (0) | 0 (0) | 0 (0) | - |
| **ACLF grade** |  |  |  |  |
| ACLF-1 | 34 | 19 | 15 |  |
| ACLF-2 | 127 | 71 | 56 |  |
| ACLF-3 | 17 | 7 | 10 |  |
| **Adverse Outcome, n (%)** |  |  |  |  |
| 28-day death | 47 (26.4) | 18 (18.6) | 29 (35.8) | 0.009 |
| ACLF-1  ACLF-2  ACLF-3 | 4  30  13 | 2  11  5 | 2  19  8 |  |
| 90-day death | 73 (41.0) | 31 (32.0) | 42 (51.9) | 0.011 |
| ACLF-1 | 7 | 3 | 4 |  |
| ACLF-2 | 52 | 23 | 29 |  |
| ACLF-3 | 13 | 5 | 8 |  |

IQR, interquartile range; Other hepatitis virus means co-infection with hepatitis A virus, hepatitis C virus or hepatitis E virus.

**Supplementary Table 3. Characteristics of patients without cirrhosis between HBV reactivation and non-reactivation**

|  | Total (n=318) | Non-HBV  Reactivation(n=47) | HBV Reactivation (n=271) | P value |
| --- | --- | --- | --- | --- |
| **Demographic, n (%)** |  |  |  |  |
| Male sex | 266 (83.6) | 40 (85.1) | 226 (83.4) | 0.94 |
| Age, *y*, mean±SD | 39.8 [31.6;46.8] | 39.2 [34.0;47.1] | 39.8 [31.1;46.8] | 0.69 |
| Alcohol consumption | 22 (6.9) | 5 (10.6) | 17 (6.27) | 0.34 |
| Other hepatitis virus | 9 (2.83) | 1 (2.13) | 8 (3.0) | 1.00 |
| **Complication, n (%)** |  |  |  |  |
| Bacterial infection | 20 (6.29) | 7 (14.9) | 13 (4.80) | 0.02 |
| Ascites | 28 (8.81) | 5 (10.6) | 23 (8.49) | 0.58 |
| Gastrointestinal bleeding | 0 (0) | 0 (0) | 0 (0) |  |
| Hepatic encephalopathy |  |  |  | 0.15 |
| Grade I | 1 (0.31) | 1 (2.13) | 0 (0.00) |  |
| Grade II | 0 (0) | 0 (0) | 0 (0) |  |
| Grade III | 0 (0) | 0 (0) | 0 (0) |  |
| Grade IV | 0 (0) | 0 (0) | 0 (0) |  |
| **HBV markers** |  |  |  |  |
| HBeAg positive, n/N (%) | 202/291(69.4) | 24/43 (55.8) | 178/248 (71.8) | 0.03 |
| HBeAb positive, n/N (%) | 65/290 (22.4) | 8/42 (19.0) | 57/247 (23.1) | 0.23 |
| HBV DNA, *log_10_ IU/ml*, | 6.02 [4.59;7.23] | 2.67 [1.67;3.84] | 6.42 [5.38;7.42] | <0.001 |
| **Organ failures, n (%)** |  |  |  |  |
| Liver failure | 85 (26.7) | 17 (36.2) | 68 (25.1) | 0.16 |
| Coagulation failure | 8 (2.52) | 3 (6.38) | 5 (1.85) | 0.01 |
| Kidney failure | 1 (0.31) | 0 (0.00) | 1 (0.37) | 1.00 |
| Cerebral failure | 2 (0.63) | 0 (0.00) | 2 (0.74) | 1.00 |
| **Adverse Outcome, n (%）** |  |  |  |  |
| 28-day death | 8 (2.5) | 3 (6.4) | 5 (1.8) | 0.10 |
| 90-day death | 17 (5.4) | 5 (10.6) | 12 (4.4) | 0.149 |
| Diagnosed as ACLF within 28d | 36 (11.3) | 10 (21.3) | 26 (9.6) | 0.037 |

Note: HBV DNA was presented as median (IQR); SD, standard deviation; IQR, interquartile range. The identification of organ failure was based on CLIF-OF.
